# Contraceptive Use in Females With Advanced CKD: A Qualitative Study

**DOI:** 10.1016/j.xkme.2023.100738

**Published:** 2023-10-10

**Authors:** Silvi Shah, Goni Katz-Greenberg, Priyanka Gudsoorkar, Prema Vyas, Sunshine Barhorst, Prasoon Verma, Meredith Pensak

**Affiliations:** 1 Division of Nephrology, Department of Internal Medicine, University of Cincinnati, Cincinnati, OH; 2 Division of Nephrology, Department of Medicine, Duke University School of Medicine, Durham, NC; 3 Department of Environmental Health, University of Cincinnati, Cincinnati, OH; 4 Burnett School of Medicine at Texas Christian University, Fort Worth, TX; 5 Department of Pediatrics, University of Cincinnati College of Medicine, Cincinnati, OH; 6 Division of Neonatology, Cincinnati Children’s Hospital Medical Center, Cincinnati, OH; 7 Department of Obstetrics and Gynecology, University of Cincinnati, Cincinnati, OH

**Keywords:** Contraception, dialysis, female health, kidney disease, perspectives, transplant, women

## Abstract

**Rationale & Objective:**

Pregnancy in females with kidney disease is not uncommon and is associated with adverse maternal and fetal outcomes. The use of contraception in females with chronic kidney disease remains low. We sought to describe the perspectives of female patients with advanced chronic kidney disease on the use of contraception.

**Study Design:**

Qualitative study.

**Setting & Participants:**

We conducted 5 focus group interviews involving 16 adult female patients with advanced chronic kidney disease (n = 3 nondialysis nontransplant chronic kidney disease, n = 9 kidney transplant, and n = 4 kidney failure receiving dialysis) in the United States, following which thematic saturation was reached.

**Analytical Approach:**

Interview transcripts were analyzed thematically.

**Results:**

We identified the following 5 themes: 1) variable knowledge regarding reproductive health with kidney disease, 2) inadequate counseling about contraceptive use, 3) lack of interdisciplinary coordination regarding contraceptive use, 4) insufficient educational resources available to guide the contraceptive discussion, and 5) need for research to better understand reproductive needs in females with kidney disease.

**Limitations:**

Patients were from a single center in the United States, and the study is limited by the transferability of findings to other settings.

**Conclusions:**

Patients with chronic kidney disease report emotional challenges with reproductive health, lack of counseling and care coordination, and insufficient resources for contraceptive use. Strategies to strengthen these factors may improve the quality of reproductive care and increase contraceptive use for females with chronic kidney disease.

## Plain-Language Summary

Pregnancy in females with kidney disease is common and associated with a higher risk of adverse maternal and fetal outcomes, but the use of contraception remains low. Little is known about female patients’ experiences in contraceptive use that may contribute to low contraceptive use in this high-risk population. In the present study using focus group interviews, patients with chronic kidney disease reported emotional challenges with reproductive health, lack of counseling and care coordination, and insufficient resources for contraceptive use. Interventions are needed to strengthen these factors to improve the quality of reproductive care and increase contraceptive use for females with chronic kidney disease.

Editorial, XXX

Chronic kidney disease (CKD) affects up to 3% of females during their childbearing years, presenting unique and challenging needs to reproductive health care for this population.^1^ Although CKD is associated with reduced fertility, pregnancy in patients with CKD is not uncommon.^2^ History of CKD increases the risk of adverse pregnancy outcomes, including pre-eclampsia, gestational hypertension, low-birth-weight infant, and preterm births.^3^ Additionally, females with kidney disease are frequently on teratogenic medications, such as lisinopril and mycophenolate mofetil, which increase the risk of miscarriages.^4^ Therefore, safe and effective contraception should be readily available, especially to females with kidney disease who wish to delay or avoid pregnancy.

Unplanned pregnancies are common worldwide in females with kidney disease and have been reported to range from 30%-50%.^5^^,^^6^ Given the medical, ethical, and emotional complexities of pregnancy with CKD, it is paramount that pregnancies are planned in this high-risk population. Contraceptive counseling is increasingly emphasized as a critical aspect of comprehensive health care to optimize reproductive outcomes. ^4^^,^^7^ However, it is not common practice for kidney-specialist health care providers to discuss contraception with their patients.^8^ As such, contraceptive use among females with kidney disease remains low at 5%-10% compared with 60% in the general population.^9^^,^^10^ Little is known about female patients’ experiences of their engagement and participation in contraceptive use that may contribute to low contraceptive use in this high-risk population.^11^

Currently, the approach to contraceptive counseling in patients with CKD is widely variable. With an increasing focus on shared decision making, the clinical practice should involve explicit consideration of female patients’ values.^12^ To perform an optimal intervention, we must understand patient perspectives and barriers to contraceptive use. The study aimed to describe the beliefs and experiences regarding contraceptive use in females with advanced kidney disease to inform reproductive health counseling, prevent unintentional pregnancies, and improve health and psychosocial outcomes.

## Methods

### Study Population and Participant Selection

Potential participants were recruited by clinicians practicing at the University of Cincinnati from outpatient nephrology, kidney transplant, and gynecology clinics. Clinicians identified patients as eligible if they identified as a woman of childbearing age with a history of kidney disease. Participants provided informed consent for participation in the qualitative study, which included a physician chart review and the digital recording of the focus group sessions. All participants were given an honorarium of $50 (clin card) for their participation.

### Data Collection

We conducted 5 focus groups with 16 participants virtually via Zoom. Each focus group comprised 2-4 participants. Focus groups were conducted until thematic saturation was reached. Each focus group lasted approximately 60 minutes in length. Each focus group was led by the principal investigator (SS) or trained moderator (PG). The question guide was developed based on a literature review and team discussions (Item S1). Questions aimed to explore perceptions, barriers, motivators, and outcome expectations for the use of contraception in females with CKD. The principal investigator or the trained moderator led the focus group discussions, asking 8 open-ended questions to assess participants' knowledge, experience, perceived barriers to contraceptive use, and potential interventional strategies that might be acceptable to them and sustainable. Participants provided information on topics related to decision making for the type of contraception, including but not limited to, whom they would like to decide, the most useful format of risk and benefit information, fear and concern with the contraceptive use, and any unmet information needs. All focus groups were digitally audio-recorded and transcribed using the using the “simple transcript” guidelines.^13^

### Statistical Analysis

All identifying personal information was removed before content analysis. Two research team members independently developed a coding scheme representing the relevant concepts addressed during the discussions. Using thematic analysis, the qualitative consultant assisted the principal investigator in reading the transcripts line by line, identifying key concepts, themes, and representative quotes, and developed a preliminary coding scheme to determine participants’ reasons for choices. Similar concepts were grouped into themes and subthemes, which emerged during the focus group discussions. Reaching thematic saturation allowed us to reduce overlap and redundancy among the categories. The revised themes and transcripts were entered into HyperRESEARCH software (version 3.7.5; ResearchWare, Inc.). The preliminary findings were discussed among the research team members, who were given 2 weeks to provide additional perspectives (member checking). Feedback was integrated into the analytical framework. The University of Cincinnati institutional review board approved the study (approval number: 2021-1065) and followed the Consolidated Criteria for Reporting Qualitative Health Research.^14^

## Results

### Baseline Demographics and Clinical Characteristics

Sixteen female patients participated in the study. The median age at the time of the study was 36 years (interquartile range [IQR], 35.25-38.75). Of the 16 patients, 10 (62.5%) were White, and 6 (37.5%) were Black. Diabetes was the cause of CKD in 6 (37.5%) of the participants, with 5 (31.25%) having documented glomerulonephritis, 2 (12.5%) with hypertension, and 3 (18.75%) having ‘other’ as the cause of their CKD. Nine (56.25%) patients in our cohort were kidney transplant recipients, 3 (18.75%) had nondialysis nontransplant CKD, and 4 (25%) were receiving maintenance dialysis, of which 2 were receiving home dialysis (1 patient with home hemodialysis and 1 patient peritoneal dialysis). Regarding kidney function, eGFR ranged from 31-97 mL/min/1.73 m^2^ for kidney transplant recipients and 23-80 mL/min/1.73 m^2^ for nondialysis nontransplant patients with CKD. Demographic and clinical characteristics are shown in Table 1.

**Table 1:** Participant Demographics and Clinical Characteristics (N = 16)

Patient Characteristics	N (%)
Median age, y (range)	36 (26-44)
Race/ethnicity	
White	10 (62.50)
Black	6 (37.50)
Cause of kidney disease	
Diabetes mellitus	6 (37.50)
Glomerulonephritis	5 (31.25)
Hypertension	2 (12.50)
Others	3 (18.75)
Type of chronic kidney disease	
Nondialysis nontransplant chronic kidney disease	3 (18.75)
Kidney transplant	9 (56.25)
Kidney failure receiving dialysis	4 (25.00)

### Themes

**Figure 1:**
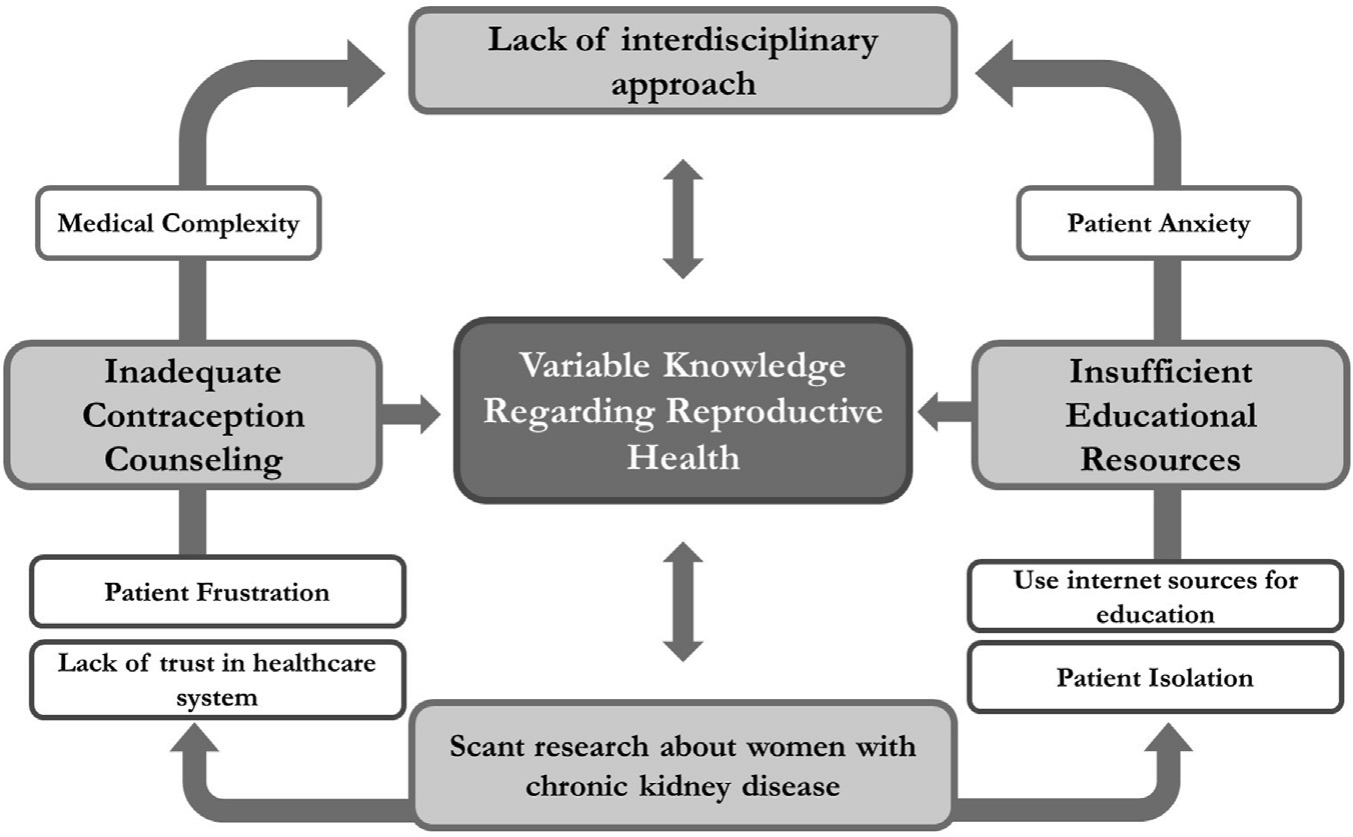
Thematic schema illustrating the relationship between barriers and attitudes of females with kidney disease and their knowledge regarding kidney disease and reproductive health.

We identified the following 5 major themes: 1) variable knowledge regarding reproductive health, 2) inadequate counseling about contraceptive use, 3) lack of interdisciplinary coordination regarding contraceptive use, 4) insufficient educational resources available to guide the contraceptive discussion, and 5) need for research to better understand reproductive needs in females with kidney disease. The themes and respective subthemes are described in the following sections, with illustrative quotations provided in Table 2. A thematic schema showing the patterns and relationships among themes and the attitudes and barriers of the participants is provided in Fig 1.

**Table 2:** Illustrative Quotations by Themes

Theme	Representative Quotations
**Variable knowledge** *Patient frustration* *Critical timing*	*"I suspect that kidney issues cause fertility issues just from my own personal experience, but I have not had a doctor come right out and tell me that so far*.*” (32, dialysis)* *“I was nervous about intrauterine device because of possible infection with my immune system being compromised, so I did not go that route*.*” (43, kidney transplant)* *“Pretty much the doctors do not tell you anything except for if you bring it forward and you know something about it already to be able to ask and to direct them as to what you want to do and all that.” (35, kidney transplant)* *"I just really wish someone explained how kidney disease affects fertility because I think it would have set me up for a little bit less heartache when things came up and things were not so easy*.*” (32, dialysis)*
**Inadequate contraception counseling** *Denied access* *Medical complexity* *Lack of thoroughness*	*“My regular gynecologist and endocrinologist are the only ones who really talked about hormones and reproductive health*.*” (43, kidney transplant)* *“I have not received anything (counseling on contraception). I think when I originally was on dialysis, I was told that my overall health with it could be delayed or kind of non-existent wanting to be active and so you know but no counseling on it nothing*.*” (39, kidney transplant)* *“I lost my child in 2011*. *After all that, I tried to go to a Planned Parenthood to get on some type of birth control myself because I am in renal failure there is nothing they will give you, nothing.” (36, dialysis)* *“I did not even know you can get contraceptives while on kidney failure because they tell you, you cannot get pregnant, or you are going to die if you do and just leave it there like I never knew that. This study interested me because I did not know we were offered contraceptives.” (38, dialysis)* *“I think the biggest issue with contraceptives is knowing which one will work with the medicine that I take because we have to be very careful*.*” (39, kidney transplant)* *“They assume that you should already know like do not get pregnant because you are on dialysis*. *OK, give me more detail on what could happen. They just need to do better with informing us as patients on the pros and cons of what could happen if you get pregnant during dialysis while you are in this chapter of your life, and they do not.” (36, dialysis)*
**Lack of interdisciplinary approach** *Patient confusion* *Fragmented care* *Inadequate physician training*	*“When I asked my gynecologist about certain things, like hair loss with medroxyprogesterone, she said to ask my kidney doctor, and when I asked my kidney doctor, they say ask your gynecologist. This can be confusing.” (43, kidney transplant)* *“My doctor said go and talk to your gynecologist and when I did, he pretty much said you are screwed*.*” (35, kidney transplant)* *“Everything is very sectioned off you know nephrology takes care of this, obstetrics takes care of this, nobody dare they ever you know cross communicate.” (32, kidney transplant)* *“My nephrologists and gynecologist are in different networks and so from my perspective I do not think that they coordinate or discuss at all. I think I am the intermediary.” (30, kidney transplant)* *“The more I think about this the more I wonder if they (doctors) are staying quiet because they do not have the schooling or the information themselves to give so to say nothing is better for them*.*” (35, kidney transplant)*
**Insufficient educational resources** *Use of internet sources* *Support groups* *Mistrust in medical system*	*“I feel very uninformed, so I went down a rabbit hole researching last night and now I am freaking out*.*” (36, CKD)* *“Kidney disease Facebook groups, just talking to other people going through it, that helped a lot in terms of hearing their experiences and then sort of you know asking Doctor Google*.*” (36, kidney transplant)* *“I do not remember much education helping us choose what would be best for our bodies, and I think it was just do not get pregnant, …you cannot get pregnant on cellcept*.*” (30, kidney transplant)* *“I had heard through some Facebook groups with kidney disease and stuff, other people sharing their stories of just the hardships that have gone on with their bodies during pregnancy or their concerns about it.” (36, kidney transplant)* *“I do not trust that I would get the right education and ahead of time unless if I went and found it somewhere else, pretty much Google in other words*.*” (35, kidney transplant)*
**Need for research** *Patient isolation* *Medical assurance*	*“I think you know research certainly. You know, maybe there do need to be different methods that are better for people with kidney disease than what is kind of the regular and the standard.” (30, kidney transplant)* *“It's really research, dialogue, education and people taking the time to then whittle that conversation down to you individually instead of treating you like a whole or lumping you and your group.” (43, kidney transplant)* *“I think it is really exciting that someone is finally paying attention to us a little bit because I think we are complicated and we do not want to be, but we are.” (32, kidney transplant)* *“Everybody wants to cure all the other diseases and help everybody else what about us*.” (38, dialysis)
**Positive counseling experiences**	*“I know my nephrologists were really good about talking about contraception and like hammering into my head why it's really important to be in communication and plan.” (32, kidney transplant)* *“My team actually spoke with my gynecologist, and they went back and forth about the best methods and what would be best for me with the medicines I was on, so it was pretty good.” (39, kidney transplant)* *“The team I have now is really great with communicating back along with each other whether I am in the hospital whether I'm in the center no matter where I am it gets sent back to my doctors all my doctors*. *So kidney doctor, primary care physician, obstetrician/gynecologist, everyone gets a notification about everything, which is great. I feel like if I would have had that earlier again before my hysterectomy, that would be also a great thing because that way again I would have just had a lot more support from knowing what was good and what wasn't*.*” (36, dialysis)*

### Variable Knowledge Regarding Reproductive Health

#### Patient Frustration

Participants shared that their knowledge regarding reproductive health with kidney disease teams based on their experience or research, not from counseling by health care providers. Patients express frustration and “heartache” caused by a lack of knowledge regarding fertility and reproductive health.

#### Critical Timing

Respondents state that they wish their physician had brought up the conversation of contraception and fertility earlier during their interactions. They also feel that physicians do not discuss contraception and pregnancy unless they bring it forward. The timing of education is critical to patients, so that they can address their family planning goals.

### Inadequate Counseling About Contraception

#### Medical Complexity

Respondents report needing more counseling regarding their reproductive health from their nephrologists. They state that “endocrinologists and gynecologists” try to counsel them; however, patients are still hesitant to follow their guidance due to the complexity of their kidney disease. They report not knowing which one works with their immunosuppressants and are reluctant to add new medications.

#### Denied Access

Participants also stated that they were never offered contraceptives and were unaware they should be using contraception. Others expressed being denied contraception, even when the patient attempted to obtain contraception herself. One participant shared how she tried to get contraception after having a miscarriage to prevent that from occurring again but was denied.

#### Lack of Thoroughness

Quality of counseling is a recurring subtheme; participants express wanting more “detail” and “pros and cons” related to contraception. Participants emphasize knowing the dangers of getting pregnant while getting dialysis or during kidney failure. Still, they lack thorough counseling to understand why they need contraception and the best contraceptive methods given their kidney disease.

### Lack of Interdisciplinary Coordination

#### Patient Confusion

The lack of interdisciplinary coordination is a clear theme that emerges. Participants repeatedly report that “everything is sectioned off” and that there is “no communication” between their gynecologist and nephrologist. Due to this lack of coordination, participants report feeling “confused.” They also discuss how they receive different answers and responses from providers, increasing confusion.

#### Fragmented Care

When discussing interdisciplinary care, patients describe their care as “sectioned off” without coordination. Patients feel like an “intermediary” and need to coordinate discussions themselves because physicians do not “cross-communicate” with each other. They report that physicians only address concerns about their specialty and do not move beyond their specialty.

#### Inadequate Physician Training

Patients also question whether all physicians have the appropriate training to educate patients about contraception and kidney disease. They express that physicians' lack of training in women’s health could contribute to the poor interdisciplinary approaches and patient education.

### Insufficient Educational Resources

#### Use of Internet Sources

Participants reiterated that a lack of educational resources forces them to turn to search Google and the internet, which can lead to the spread of misinformation. This can also cause further frustration and anxiety.

#### Reliance on Support Groups

Many participants express going to kidney disease Facebook groups to learn about contraception and pregnancy. This is a place where females with kidney disease can share their stories and hardships and learn about fertility related to kidney disease.

#### Mistrust in Medical System

Participants report inadequate information about contraceptive use, which affected their decision making and choice regarding the most appropriate contraception in the context of kidney disease. Participants express that when discussing pregnancy and kidney-health, physicians highlight the importance of preventing pregnancy because of immunosuppressants and their effects, but not how to prevent pregnancy. We also see a reoccurrence of a lack of trust in physicians and health care providers to educate patients about contraception, fertility, and reproductive health in patients with kidney disease.

### Need for Research to Better Understand CKD in Females

#### Patient Isolation

Participants were asked, “What steps do you think will guide to increase the awareness of contraception with kidney disease?” They expressed that more research in reproductive health and kidney disease would help. They have stated, “What about us” and described feeling isolated and forgotten by the research community.

#### Medical Assurance

Participants believed that the research needs to be better distributed to females with kidney disease, so that they can make informed decisions regarding their reproductive and sexual health. They also express a need for research specific to females with kidney disease instead of treating them like “the standard.” They also hope the counseling is geared to each individual need rather than “lumping” patients into groups.

## Discussion

Among reproductive-aged females with advanced CKD, our qualitative study using focus group interviews revealed the following themes on contraceptive use: 1) variable knowledge regarding reproductive health, 2) inadequate counseling about contraceptive use, 3) lack of interdisciplinary approach, 4) insufficient educational resources, and 5) need for further research. Interviews with participants revealed a desire for a more thorough discussion of contraception with kidney-health care providers, and patients further advocated for multidisciplinary kidney care.

Females with kidney disease are not completely informed of changes in fertility and reproductive health that are associated with kidney disease. Prior reports note females with CKD felt the information regarding their reproductive health was often unhelpful and incomplete,^15^^,^^16^ with almost half of the participants in a previous survey noting that they were unaware females can become pregnant following transplantation^17^ These needs are not solely seen in patients with kidney disease but in other chronic conditions as well. A recent survey of liver transplant candidates and recipients found that most patients consider family planning, including contraception and pregnancy-specific counseling, a high priority and an important part of their transplant care.^18^ Importantly, 96% of participants would have liked additional counseling, and a vast majority (89%) preferred in-person counseling with their transplant provider.^18^

Participants reported that kidney doctors did not provide counseling regarding contraceptive use during routine clinic visits. Although nephrologists play a major role in caring for patients with kidney disease, only 46% counsel their female dialysis patients about contraception.^19^ A study from Canada showed that 33% of physicians discussed fertility with their patients.^20^ When trying to assess the reason for the paucity of counseling, a survey disseminated to the nephrologists in the United States and Canada by the women’s health working group of Cure Glomerulonephropathy showed that >65% of participants lacked confidence in women’s health issues, with very few providing contraception counseling to their female patients with CKD (average of less than 1 patient a month).^8^ Low confidence regarding contraception seems to stem from a lack of exposure and insufficient training in women’s health, as emphasized by the study participants as well,^21^, ^22^, ^23^ and underscores the need to include women’s health training in the nephrology fellowship curriculum.

Study participants highlighted the lack of communication between nephrologists and gynecologists as one of the barriers to contraceptive use. Segmented care of female reproductive health between disciplines results in patient frustration and a lack of trust in the health care system. Hendren et al^8^ noted that approximatey 90% of nephrologists felt that interdisciplinary guidelines could improve knowledge and lead to better patient care. Multidisciplinary and team-based care have been shown to improve patient health outcomes and patient experiences with the health care system.^24^ Following kidney transplantation, most female transplant recipients experience a return of fertility within 1 year,^25^ and conception is common,^4^ which further highlights the need to improve multidisciplinary approaches in contraceptive counseling. Integrating practice changes to improve team-based models of care will benefit patients and their families and strengthen nephrologists’ and gynecologists’ ability to provide comprehensive patient-centered reproductive care.^26^

There are inadequate educational resources to guide contraception discussions. While evidence-based data regarding contraceptive use in CKD exist,^9^^,^^10^^,^^27^^,^^28^ it does not translate to adequate knowledge for patients. Lack of counseling by kidney-health professionals and a persistent lack of coordination of care between nephrologists and gynecologists contribute to barriers in the dissemination of information regarding contraception. Although data from the past decade show that most planned pregnancies in kidney transplant recipients are successful,^29^ they are often associated with an increased risk of adverse events to the mother, the fetus, and the allograft. The risk can be decreased by ensuring that all pregnancies are planned with adequate preconception education.

Research regarding kidney disease and reproductive health tends to focus on the physiologic outcomes of pregnancy and the safety of contraceptive methods.^30^ This information is often broad and does not usually address specific CKD spectrum pathology. Furthermore, the existing research does not typically incorporate patients’ preferences or needs into guidance. Emerging data show that incorporating patient perspectives and opinions into research will improve adherence and outcomes.^12^ Participants in our study echoed this desire to be part of the research that ultimately affects them.

There are potential limitations to our study. The study population was recruited from a single-site urban academic medical center; therefore, the opinions and themes do not likely represent those of all patients with CKD. In addition, we did not collect information on prior pregnancies and contraceptive use from the participants.

Based on the themes and subthemes that emerged from the interviews, we identified the following 5 domains that should be addressed to improve care regarding contraceptive use in females with kidney disease: 1) contraceptive counseling, 2) multidisciplinary care, 3) physician-patient communication, 4) patient support groups, and 5) physician training. We have outlined suggested strategies and actions for each domain based on our participant’s experiences in Table 3. We believe that addressing these domains will help attenuate the patient-reported barriers to contraceptive use.

**Table 3:** Suggested Strategies and Actions for Clinical Practice

Domain	Suggested Strategies and Actions
**Contraceptive counseling**	• Utilize shared decision making in family planning.
	• Conduct counseling sessions with patients and their spouses individually and together.
	• Ensure counseling sessions are a part of annual visit.
	• Consider insurance and cost of contraception for patients.
	• Review medication lists with patients regularly.
	• Hold kidney smart classes virtually and in person for patients regarding contraception.
	• Increase research on contraceptive counseling in females with kidney disease.
**Multidisciplinary care**	• Share notes and plan of care among nephrologists, gynecologists, and primary care physicians.
	• Increase utilization of MyChart by specialists and patients.
	• Involve pharmacists in educating patients on medication interactions between immunosuppressants and contraceptives.
	• Encourage chart review for out-of-network specialists.
**Physician-patient communication**	• Explain to patients in detail information regarding fertility and adverse pregnancy outcomes with kidney disease.
	• Discuss pros and cons of contraception with patients.
	• Determine baseline knowledge regarding reproductive health with each patient instead of assuming what a patient knows.
	• Be honest with patients.
	• Build trust with patients.
**Patient support groups**	• Facilitate support groups for patients with kidney disease to better understand how they can be supported.
	• Offer seminars for patients that include patients who have experienced pregnancy with kidney disease.
**Physician training**	• Develop educational sessions regarding contraception for nephrologists.
	• Provide continuing medical education credit courses that help nephrologists better understand fertility and chronic kidney disease.
	• Create interdisciplinary sessions with gynecologists and nephrologists to help each specialty better understand the health of females with chronic kidney disease.

In conclusion, patients with CKD report emotional challenges with reproductive health, lack of counseling and care coordination, and insufficient resources for contraceptive use. Unplanned pregnancies among patients with kidney disease are associated with increased risks of obstetric and fetal complications,^2^ underscoring the importance of accessible, safe, and effective contraception. Improved reproductive health outcomes in females with kidney disease are especially relevant now given recent changes to major Supreme Court decisions that impact reproductive choices.^31^ Importantly, our study describes patients’ experiences with contraceptive use and identifies specific patient-reported barriers to contraceptive use in females with kidney disease. Strategies to strengthen these factors and develop future interventions may improve counseling, coordination of care, and quality of reproductive care. Ultimately, this will result in an increase in contraceptive use and lower the incidence of unplanned, high-risk pregnancies in females with kidney disease.

## Supplementary Material

Supplementary File (PDF) Item S1.
