# Supplementary material for: Contraceptive Use in Females With Advanced CKD: A Qualitative Study
Source: Kidney Med. 2023 Oct 10;5(12):100738. doi: 10.1016/j.xkme.2023.100738 (PMC10684388; doi:10.1016/j.xkme.2023.100738)
Supplement: Supplementary File (PDF) — Item S1. [file mmc1.pdf]

Item S1. Interview guide and focus group questions.

1. What type of counseling regarding contraception method/use have you received in relation to kidney disease?
    a. Who provided the counseling? Can you discuss more details?
    b. Method/approach to counseling (in-person/virtual/telemedicine)
    c. How often did you receive the counseling?
    d. When was the first and last time you received counseling?
    e. Narrate the discussion (narrative)
    f. Prompts: regular nephrologist visits, obstetrician/gynecologist visits, primary care visits, wellness/annuals.

2. Why do you think contraception and related methods are important in relation to kidney diseases?
    a. How does kidney disease impacts outcomes in pregnancy?
    b. Do you know certain medications are contraindicated with pregnancy and you may be taking it with kidney disease (like lisinopril mycophenolate)?
    c. As you might be aware fertility is impacted by kidney disease. Would you be willing to share your thoughts regarding being able to conceive with kidney disease?

3. What strategies, tools, or support systems were you introduced to decide on contraception methods to be used with kidney disease?
    a. Facilitators
    b. Barriers

4. How was your decision to utilize contraception among different types of contraception methods available, guided with kidney disease?
    a. What methods of birth control/contraception have you used?
    b. Why did you choose to use this method?
    c. Why did you choose to stop this method if you are no longer using it?
    d. Perceived barriers.
    e. Availability and feasibility.

5. How would you describe the care coordination between your nephrologist and obstetrician, regarding contraception in relation to kidney disease?
    a. Do you have a nephrologist and /or obstetrician/gynecologist that you regularly see?
    b. Do you know how your consulting nephrologist and obstetrician/gynecologist ever communicate?
    c. Do you have any suggestions on how you would like them to communicate?

6. How would you like to get information about contraception in relation to kidney disease?
    a. Who would you like to get this information from?
    b. How often would you like the information?
    c. Would you like the information to be shared with your care providing team?
    d. What format would you like this information (Prompts: text, handouts, emails)?

7. What steps do you think will guide to increase awareness of contraception in relation to kidney disease?

8. What problems did you face with access to contraceptive methods of choice during the COVID -19 pandemic?
